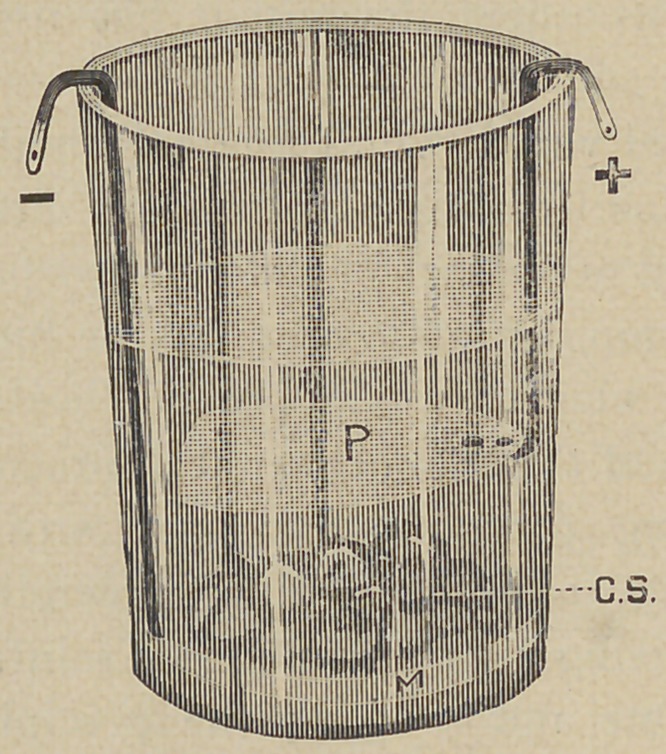# Copper Amalgam

**Published:** 1889-06

**Authors:** Levitt E. Custer

**Affiliations:** Dayton, O.


### Copper Amalgam.

BY LEVITT E. CUSTER, B.S., D.D.S., DAYTON, O.

Read before the Mississippi Valley Dental Society, Cincinnati, March, 1889.

Since in these latter days mastication begins in the kitchen, and the function of the teeth is being largely dispensed with, and since the present food stuffs are lacking in the lime salts of former days, and the teeth are not of as good structure as formerly, it is not surprising that these organs should have degenerated and have become more frequently the subject of caries. We look with horror upon the coming generation, born in wealth and affluence, which, having inherited poorly calcified teeth, shall be nourished upon foods which, in order to satisfy the cultivated palate, have given to the slop-pail those very lime salts which were needed for the more perfect formation of the osseous system and the teeth. Already the first molars of this generation are hardly erupted ere they show unmistakable signs of early degeneracy.

With such a condition of things what are we to do? It has been comparatively easy to save well calcified teeth by filling with gold or other materials, even to an artistic effect, but the conditions are changing. If we boast of being able to save all teeth at this day, in order to do this we must adopt new methods to suit the new conditions. With these poorly calcified teeth we have to look to their salvation first and artistic methods and effects afterwards. The profession has seen the results of contour fillings and porcelain filling and tips, which could be used only in the very best teeth and not in those of poor structure. We have then to resort to those methods which save teeth first, regardless of appearances; then, if possible, improve these in an artistic view.

Among the improvements made in the manufacture and manipulation of other filling materials there has been a marked one in the line of amalgams. Since the advent of the "silver paste" of Taveau over half a century ago, followed a few years later by the royal mineral succedaneum of the Crawcour Brothers, amalgam, after exciting professional dissensions out of which on the one hand grew and waxed strong, the new departure corps by which its virtues were lauded, and on the other hand, a resolution was passed by the American Society of Dental Surgeons pronouncing it mal-practice, yea, even *discreditable irregularity* to use the stuff; amalgam, after actually saving teeth which were formerly deemed beyond the power of gold, after it was found to require no little degree of art and skill to manipulate it, and after it ceased to produce mercurial poisoning whereby it formed a loop-hole for the medical empirics failures, it has finally found a legitimate place in dental practice. Not only this, but as the faults of amalgam are being more largely overcome every day in its manufacture and manipulation, its range is being gradually extended, not only as a filling material for carious teeth, but as an adjunct in crown-work and in the laboratory.

Copper has been used as an ingredient of alloys from the very first. Its value in this connection has always been acknowledged, in fact it has been the least disputed of any metal used in the manufacture of a dental alloy. When it was not used it was because of its one objection, the dark color it produced. This metal gives a saving power to the amalgam which can be attributed to no other component. The old-fashioned amalgams which contain copper are saving teeth to-day.

The virtues of copper in this relation are several. First, it prevents, or lessens the spheroiding tendency. This is due to the difficulty with which this metal is amalgamated, so that these unamalgamated surfaces act as a resistance to change of shape. Second, these unamalgamated surfaces when exposed form a salt of copper which, according to Tomes, is first a sulphide which, " under the influence of air and moisture readily becomes oxidized and forms the sulphate." This layer then of sulphate of copper there formed acts in two ways, namely, a mechanical and a therapeutic. In a mechanical manner it fills up the gap caused by the slight change of shape if any, and forms an intervening and more compatible layer between the filling and the tooth structure. Tomes goes so far as to say that " this sulphate is freely soluble, and hence is likely to permeate the dentine, when it will again be converted into a sulphide, whilst the sulphides of other metals, not being so readily converted into soluble salts, will not so thoroughly permeate the teeth." By this more compatible layer Flagg says he can fill closer to the pulp and have less shock from thermal changes. In a therapeutic relation the sulphate of copper acts as an antiseptic. Probably this is the strongest argument in its favor. As an antiseptic it prevents fermentation and the growth and action of leptothrix or bacterium lactis. Dr. Black, after twenty-six experiments with different filling materials in test tubes containing "beef-extract, sugar solution previously infected with carious fungi (pure culture)" says: "We see from these results that the only filling at present in use which exerts a continual antiferment action upon the walls of the tooth, and its immediate surroundings, is the old copper amalgam; not only that, but the very substance of the tooth containing such a filling itself becomes antiseptic, a piece of bluish or bluish-green dentine from such a tooth very powerfully retarding the development of the fungi, and indeed in two such cases completely destroying them. Secondary decay in such a case would be next to impossible where any thing like cleanliness was observed." The antiseptic power of the copper sulphate thus formed I think is accepted by every one; even Dr. St. George Elliot, of England, who lately had an article in the London *Dental Record* upon the shrinkage of pure copper amalgam, says it "is a material of considerable value as an antiseptic."

Copper as a component of an alloy or amalgam with all these good qualities is not without an objection. The very thing that makes it of value as an antiseptic, at the same time lessens its value from the artistic standpoint—the copper salts are black. A filling having copper as an ingredient turns horribly black and at times the tooth itself will become darkened by the infiltration of copper salts. But we very often have to deal with teeth of poor structure, and as first stated, with the incoming generation we shall have more of them which must be filled with a material which has more than a mechanical effect or else be crowned. The appearance is but little compared with the salvation of the tooth, especially in bicuspids and molars. The English have saved teeth for years better by the use of copper alloyed amalgams than we who have been more artistic and used a white amalgam. Dr. Black says: "I do not hesitate to say that if our only object is to check the destruction of tissue by caries, there is no material at present in use with which this object may be so surely accomplished as with the use of copper amalgam."

In view of the virtues of copper as a component of alloys, as shown and proven for fifty years, of late the effort has been made to form an amalgam composed entirely of pure copper and mercury. How far this has been successful, is the object of this paper, for as well as others the author has been working in this direction.

Copper is a metal very difficult to amalgamate. Mercury will not directly combine with it under ordinary circumstances at all. When it was found necessary for different purposes to coat copper with mercury a strong solution of mercuric nitrate was rubbed upon the surface when it would become coated with mercury. Taking advantage of this I endeavored to procure a copper amalgam by triturating precipitated copper with free mercury in a solution of mercuric nitrate. Pure precipitated copper was obtained by immersing a strip of zinc in a solution of chemically pure copper sulphate and washing the precipitate with hydrochloric acid. Copper amalgam was obtained by this process it is true, but the amount of work required for the small amount of amalgam obtained caused me to look in other directions. I thought to take advantage of the law of nascentcy of elements, when an element is in its most active condition, when one is liberated in the free state, or when changing its form, as being precipitated from a solution. About this time I saw the article of Dr. Weagant in the *Dental Review* upon the manufacture of copper amalgam by precipitating the copper from a sulphate solution of a strip of iron in a glass containing mercury. The copper comes out of the solution in direct contact with the mercury and there the two combine. Iron is used to precipitate because the mercury has no affinity for it as for zinc. But after a thorough trial of the method, while it was an improvement upon the mercuric nitrate method, the slowness of the operation and the danger of contamination with iron, lead to further study and experimentation.

The idea of decomposing a solution of copper by an electric current, or in other words, the possibilities of electrolysis had at times suggested itself, but not having the facilities had not been tried. Purchasing a four-cell common copper and zinc battery, the bottom of a glass tumbler was covered with mercury and filled with a solution of chemically pure copper sulphate. Allowing the negative wire of the battery to extend to the bottom of the glass and in contact with the mercury, this, the mercury then became the negative electrode, M. The positive wire ended in a strip of platinum which was immersed in the solution but not touching the mercury, and thus was formed the positive electrode, P.

According to the laws of electrolysis electro-negative elements go to the positive electrode and electro-positive to the negative electrode. Sulphur and oxygen being electro-negative were liberated in the form of $SO_4$, technically known as *sulphione*, at the positive electrode. Hydrogen and copper being electro-positive were liberated at the negative electrode. The hydrogen escapes as a gas and the copper being in a nascent state combines with the mercury constituting the cathode in which it seems to assume a crystalline form as can be felt by the finger.

After a little experimentation it was found that instead of renewing the solution of copper sulphate, all that was necessary was to add a few crystals of chemically pure copper sulphate which would rest upon the mercury until they were dissolved, C. S. Thus the process was continuous; as fast as the solution was decomposed fresh copper sulphate was dissolved from the crystals, and all that was necessary was to take out the mercury just where it became stiff enough to handle by being filled with copper. After this is triturated a little it will be found that probably only one-third of its bulk is copper and a large portion of the mercury may be squeezed out through the chamois skin and put back in the glass to begin again, a little more being added to take the place of that which remained in combination with the copper. So you see there is no waste of mercury.

The amount produced by this process was very encouraging. With a battery like the one above described, when in good condition, about one-half ounce of copper could be amalgamated in one night.

I now thought that I alone had reached the acme of success in the manufacture of pure copper amalgam, but like a cold wave that sweeps over a country a good many are affected at the same time, and I was one of them. When the copper amalgam question came up there were others working in that direction also, but they had kept their discoveries secret, and every one I suppose independent of the other; at least my own were entirely original with myself. I was very much chagrined, however, when at Chicago last month to find that the manufacturers of copper amalgam in that city were using a process quite similar to the one I had thought out. I found I was not the only smart one in that direction by any means. The processes differ in this respect, however; instead of using a platinum electrode they use electrolytic copper as an anode which, in a solution of dilute sulphuric acid, is dissolved by the action of sulphione, and entering the solution is liberated at the cathode, or mercuric anode. This process is simply the electro plating of mercury. The cost of chemically pure copper sulphate is about twice that of the electrolytic copper. But owing to the fact that electrolytic copper is only 98 per cent. pure, being contaminated with antimony, bismuth, and arsenic, I claim originality in presenting a method by which *absolutely* pure copper amalgam may be produced with no more trouble, and with but an additional expense of one and one-half cents per ounce.

The copper having been amalgamated the next step is to get rid of the surplus mercury, and it is by this experience that I will be enabled to give a few practical hints as to the manipulation of copper amalgam preparatory to filling. All of us have not the facilities of a hydraulic press to squeeze out the surplus mercury, so under such circumstances it can best be separated by successively heating, triturating, and squeezing out through the chamois skin. This is repeated until after setting the required density has been obtained. Herein lies the point of the edge strength of copper amalgam; the more free mercury that can be taken from the mass the more dense will be the amalgam. After three or four repetitions of the process it will be found that the product when crystallized will be as hard as ordinary amalgam. It is then ready for use and comes to you in the form of tablets or sticks.

Now in regard to manipulation. After having the pure copper amalgam the next thing for the best results is proper preparation for filling. I may add right here that the whole secret and the most important part of copper amalgam filling lies in this part of the operation. In amalgamating common alloys the all important point to have been observed was to so proportion the parts that no mercury need be squeezed out. A certain amalgam always sets in about the same time. The manipulation had very little to do with that quality. But not so with copper amalgam. The time of setting, whether quick-setting or slow-setting the edge strength, and to a large extent the color, are all controlled in this part of the operation.

The directions usually given to render a copper amalgam plastic, are to heat in an iron spoon until globules of mercury appear upon the surface, crush in a mortar and if not plastic enough add mercury. The manner of heating seems hardly worth consideration, but this is not to be overlooked. The piece should be gradually heated by being held high above the flame and all portions heated alike—not one side a bluish-brown and the other the mercury just appearing. To do this a very good way is used by Dr. Ames, of Chicago, who holds the piece in a pair of foil pliers, revolving it over the flame. My method has been to roll my amalgam in thin wafers when first making it so that holding the piece in an old mirror frame it becomes more evenly heated than if it were a compact mass.

The amount of heat is the most important point to be observed. I found when making the amalgam in extracting the mercury that upon the amount of heat depends the time of setting as well as to some extent the future color of the filling. You can have it quick-setting or slow-setting just as you wish and at the same time have the same degree of plasticity; and for different kinds of work this is a valuable quality. Here is the point to be observed; the setting time of a pure copper amalgam is in an inverse ratio to the amount of heat applied. In other words, if you desire it quick-setting use the least amount of heat that will bring the mercury to the surface; or if you wish it slow-setting, continue the heat until the edges begin to turn a bluish brown color. All grades of setting may be obtained between these two extremes.

In regard to the color if too much heat is applied the filling will become black. I believe this to be due to the mercury being vaporized in such quantities as to leave free surfaces of copper which do not again become amalgamated during trituration. You will remember that it was the free surfaces of copper in the common copper amalgams which formed the black salts.

After the piece is heated as desired, it is then triturated in a mortar until the crystalline form is entirely broken up, and the result is either a fine powder or in a plastic condition. If it can not be made plastic by continued trituration and working in the warm palm of the hand a few globules of mercury may be added from the holder. Another method is practiced by Dr. Ames, of Chicago, which is to add in the mortar a weak solution of mercuric nitrate, one to five of water, and continue trituration. Mercury from the solution amalgamates the non-amalgamated surfaces of the copper and it immediately becomes plastic. He then washes it in a solution of bicarbonate of soda to neutralize the acidity. I find that if the alkaline solution is added before the mercuric nitrate is poured off, more mercury is precipitated and the mass becomes still more plastic. This, however, may not be needed at all times. Dr. Ames claims that by treating in this manner the resultant filling will also be whiter. My own experience bears him out in this. I believe it is due to more perfect amalgamation which leaves fewer copper surfaces exposed to form salts. While this increases the artistic effect of the filling, I think it is a question whether it is not at the expense of the saving power of the same, both in controlling the spheroiding tendency and in preventing the formation of copper salts for which virtues we are using the copper amalgam. When mercury is added from the mercuric nitrate solution it produces more perfect amalgamation. The nearer perfect the amalgamation of any amalgam the more is the tendency to spheroid. It was the free surfaces of copper in the old copper alloyed amalgams which retarded the spheroiding tendency. Also mercury added from the nitrate solution by producing more perfect amalgamation, allows less copper directly exposed to form salts. Hence if there are no salts formed there will be no intervening and more compatible layer between the filling and the tooth—nothing to fill up the space made by change of shape, and nothing to produce the antiseptic effect. Now if free mercury is added from the holder it unites with the mercury already in the amalgam and not with the copper thereby producing plasticity in that manner.

The amalgam thus made plastic is manipulated in filling in the same manner as any other amalgam, but as it can be made quick-setting can often be finished at the same sitting.

In regard to the color of a pure copper amalgam, contrary to what we would naturally expect, such a filling does not turn as dark as the old copper alloyed amalgam. This is undoubtedly due to the fact that in the old fillings as copper can not be amalgamated by trituration alone, it was not amalgamated at all; whereas, in the pure copper amalgam, although consisting of nothing but copper and mercury, in this case the copper has been almost, if not at times, completely amalgamated by the electrolytic process, and hence there are less free copper surfaces exposed to form black salts. A pure copper amalgam filling may be kept white on its exposed surface for a time by coating the surface at the next sitting with a very plastic facing amalgam of gold, silver, or tin. It will not do to use a pure copper amalgam as the base of a filling and face with a white amalgam which contains platinum and zinc, as the latter does not harden when in contact with the copper amalgam. Am happy to say I recently refilled the last of those which gave me this experience. Dr. Sillito used with success Mood's "gun-powder" amalgam for facing purposes which consists of gold, tin, and silver.

A pure copper amalgam is valuable over a copper alloyed amalgam because it is said not to spheroid, and because none but copper salts are formed, and because the waste can be used over. Elliot, of England, says it shrinks more than a common amalgam, but this as well as that it does not spheroid remains yet to be proven. The saving power of a pure copper amalgam over one which is alloyed with copper can only be proven by time.

The practical points in the maufacture, manipulation and effects of pure copper amalgam may be summed up in the following: First, the density or edge strength is in proportion to the amount of amalgamated copper; or in other words, to the extent to which the copper has been amalgamated and the surplus mercury driven out; second, the time of setting is in an inverse ratio to the amount of heat applied; third, the color becomes whiter in proportion to the degree of amalgamation; fourth, since the virtue of copper amalgam lies largely in the effect of the copper salts formed, acting in a mechanical and therapeutic manner, the saving power of such a filling is in proportion to the amount of salts formed; therefore if we endeavor to improve the appearance of a pure copper amalgam, *per se*, we defeat the object for which we are using it; fifth, as Dr. Black says there is no better filling for saving teeth than copper amalgam about which copper salts are formed, for patients of moderate circumstances and in teeth of poor structure we must at times use it regardless of an inartistic effect for its worth and not its appearance.

## Figures and Tables

**Figure f1:**